# Mitochondrial metabolism regulates macrophage biology

**DOI:** 10.1016/j.jbc.2021.100904

**Published:** 2021-06-23

**Authors:** Yafang Wang, Na Li, Xin Zhang, Tiffany Horng

**Affiliations:** School of Life Science and Technology, ShanghaiTech University, Shanghai, China

**Keywords:** mitochondrial metabolism, macrophage activation, macrophage biology, mitochondrial dysfunction, oxidative stress, α-KG, α-ketoglutarate, ΔΨm, mitochondrial membrane potential, ACLY, ATP citrate lyase, AMPK, AMP-activated protein kinase, AST, aspartate aminotransferase, CIC, mitochondrial citrate carrier, DAMPs, damage-associated molecular patterns, Drp1, dynamin-related protein 1, DMF, Dimethyl fumarate, ETC, electron transport chain, eIF5A, hypusination of translation factor eukaryotic initiation factor 5A, ER, endoplasmic reticulum, FAO, fatty acid oxidation, FADH_2_, flavin adenine dinucleotide hydride, GABA, γ-amino butyric acid, GAD, glutamic acid decarboxylase, GAPDH, glycolytic enzyme glyceraldehyde 3-phosphate dehydrogenase, GSDMD, gasdermin D, HIF-1α, hypoxia inducible factor 1α, HMGB1, high mobility group box 1, IDH, isocitrate dehydrogenase, IFNγ, interferon-γ, IL-4, interleukin-4, iNOS, inducible nitric oxide synthase, IRG1, Immune-Responsive Gene 1, KLF4, Krüppel-like factor 4, LPS, lipopolysaccharide, MAS, malate-aspartate shuttle, MDVs, mitochondria-derived vesicles, MHC, Major Histocompatibility Complex, mTOR, mammalian target of rapamycin, mtROS, mitochondrial reactive oxygen species, NADH, adenine diphosphate hydride, NF-κB, nuclear factor kappa B, NLRP3, NOD-, LRR- and pyrin domain-containing 3, NO, nitric oxide, OAA, oxaloacetate, oxPAPC, 1-palmitoyl-2-arachidonyl-sn-glycero-3-phosphorylcholine, OXPHOS, oxidative phosphorylation, PAMPs, pathogen-associated molecular patterns, PARP, Poly (ADP-ribose) polymerase, PGE2, Prostaglandin E2, PHD, prolyl hydroxylases, PPAR, peroxisome proliferator-activated receptor, RET, reverse electron transport, SDH, succinate dehydrogenase, SIRT, Sirtuin, SUCLG1, succinyl-CoA synthetase, SUCNR1, succinate receptor 1, TCA, tricarboxylic acid, TLR, Toll-like receptor, UPR, unfolded protein response

## Abstract

Mitochondria are critical for regulation of the activation, differentiation, and survival of macrophages and other immune cells. In response to various extracellular signals, such as microbial or viral infection, changes to mitochondrial metabolism and physiology could underlie the corresponding state of macrophage activation. These changes include alterations of oxidative metabolism, mitochondrial membrane potential, and tricarboxylic acid (TCA) cycling, as well as the release of mitochondrial reactive oxygen species (mtROS) and mitochondrial DNA (mtDNA) and transformation of the mitochondrial ultrastructure. Here, we provide an updated review of how changes in mitochondrial metabolism and various metabolites such as fumarate, succinate, and itaconate coordinate to guide macrophage activation to distinct cellular states, thus clarifying the vital link between mitochondria metabolism and immunity. We also discuss how in disease settings, mitochondrial dysfunction and oxidative stress contribute to dysregulation of the inflammatory response. Therefore, mitochondria are a vital source of dynamic signals that regulate macrophage biology to fine-tune immune responses.

Macrophages safeguard tissue homeostasis and regulate inflammatory responses. To exert these varied functions, macrophages show high plasticity and adopt different activation states according to the stimulus signals. The Th1 cytokine interferon-γ (IFNγ) together with Toll-like receptor (TLR) ligands, including lipopolysaccharide (LPS), promotes classically activated proinflammatory macrophages (commonly known as M1-like macrophages), which secrete proinflammatory cytokines such as interleukin-6 (IL-6) and IL-1β to induce inflammatory responses and fight against infection; generate highly reactive oxygen species and nitrogen intermediates to gain efficient microbicidal and tumoricidal activities; and increase major histocompatibility complex (MHC)-I/II, CD80, and CD86 expression (1, 2). However, continuous and excessive activation of proinflammatory macrophages may lead to sustained inflammation and accessory tissue damage (3). Macrophages can also be activated by other stimulating factors to alternatively activated states. For example, the Th2 cytokines interleukin-4 (IL-4) and IL-13 induce macrophage alternative activation (commonly known as M2-like activation) (3, 4). These macrophages attenuate Th1/M1-driven inflammation, facilitate tissue repair and remodeling, and induce Th2-driven pathologies, such as asthma and helminth infections. Such macrophages highly express a range of specific scavenging molecules, including mannose and galactose receptors and enzymes such as arginase (5, 6). In response to various kinds of environmental stimuli, macrophages populations will change their physiology and shift their phenotype, which allow them to actively participate in disease resolution or progression (7).

Recent studies indicate that shifts in mitochondrial metabolism and physiology are vital for macrophage activation to different states, including alterations of oxidative metabolism, mitochondrial reactive oxygen species (mtROS), tricarboxylic acid (TCA) cycle, mitochondrial ultrastructure, and membrane potential. The signals that drive macrophage inflammatory activation induce breaks in and rewire the TCA cycle by influencing expression of TCA cycle enzymes, IDH (isocitrate dehydrogenase) and SDH (succinate dehydrogenase), resulting in elevations in citrate and succinate, respectively. These signals also augment glycolysis (also known as Warburg Effect). In contrast, IL-4-activated macrophages maintain an unbroken TCA cycle and preferentially engage oxidative phosphorylation (OXPHOS) and fatty acid oxidation (FAO) for ATP production. OXPHOS in IL-4-activated macrophages is fueled by the oxidation of fatty acids and glutamine, which activates the peroxisome proliferator-activated receptor-γ (PPARγ) to mediate the induction of genes regulating alternative macrophage functions (8). Glucose oxidation, induced by the mTORC2-IRF4 signaling axis, also contributes to IL-4 mediated gene induction (9).

Shifts in mitochondrial metabolism are closely linked to macrophage activation. In this review, we discuss the mechanistic underpinnings of differential mitochondrial metabolism in distinct macrophage activation states, discuss how they are induced and how they contribute to macrophage activation and biology.

## Macrophage activation signals regulate shifts in mitochondrial metabolism

The type I inflammatory response usually starts when macrophages and other sentinel cells are activated by pathogen-associated molecular patterns (PAMPs), including microbial cell wall components, nucleic acids, and lipoproteins. Macrophage metabolism also undergoes dynamic changes during such activation. At the center of cellular metabolism is the mitochondria, which not only supplies energy but is also involved in biosynthesis and maintaining cellular redox and serves as a platform for various innate immunological signaling pathways (10). Macrophage activation signals alter the activity of the electron transport chain (ETC) and the TCA cycle to influence multiple aspects of mitochondrial metabolism. They also induce an upregulation of glucose and glutamine utilization and a shift toward anabolic pathways. Aerobic glycolysis, induced by LPS-stimulated mammalian target of rapamycin (mTOR) and hypoxia-inducible factor 1-alpha (HIF-1α) pathways (11), is upregulated for ATP production while OXPHOS is repressed through multiple mechanisms, including the two breaks in the TCA cycle. One break results from decreased expression of IDH, the TCA cycle enzyme that converts citrate to α-ketoglutarate (α-KG), allowing for the cumulation of citrate, which can be redirected for generating itaconic acid or withdrawn for fatty acid biosynthesis (12). The second break occurs after succinate, with a novel pathway termed the aspartate-arginosuccinate shunt, which can produce arginine to support nitric oxide (NO) production. NO generated by inducible nitric oxide synthase (iNOS) can hamper mitochondrial respiration and impair the plasticity of proinflammatory to anti-inflammatory repolarization, and LPS plus IFNγ stimulation can inhibit FAO (13). Consistently, oxidative metabolism is suppressed in LPS-tolerant macrophages, which are no longer able to produce inflammatory cytokines as a result of long-term LPS exposure (14). Note that while some characteristics of tolerant macrophages resemble that of the M2 macrophages, it would be an oversimplification to equate the two macrophage states, which differ in many aspects of metabolism, phenotype, and function (14).

In contrast, IL-4-activated macrophages have more demand for glucose, glutamine, and fatty acids compared with inflammatory macrophages and rely on β-oxidation. The increased fatty acid necessary for engaging mitochondrial OXPHOS is derived from lipolysis of triglycerides (15). This metabolic adaptation results in a shift in the proportion of NADH and FADH_2_ (nicotinamide adenine dinucleotide and flavin adenine dinucleotide) that feeds the ETC (16). Inhibition of FAO is sufficient to repress the alternatively activated macrophage phenotype and induce M1-like gene programs (17).

Similar to PAMPs, self-encoded damage-associated molecular patterns (DAMPs) such as mitochondrial DNA and N-formyl peptides (NFP) are detected by macrophages leading to induction of inflammatory responses (18). Recent studies indicate that a class of DAMPs represented by oxidized naturally occurring phospholipids, derived from 1-palmitoyl-2-arachidonyl-sn-glycero-3-phosphorylcholine (PAPC) and collectively known as oxPAPC, resides in cell membranes and lipoproteins and functions together with PAMPs to induce optimal immune responses (19, 20). OxPAPC modulates cell metabolism by upregulating mitochondrial respiration and OXPHOS as well as glutamine utilization, an energy and anaplerotic carbon source that replenishes TCA cycle intermediates, leading to increased cytoplasmic levels of oxaloacetate (OAA). HIF-1α is a key transcription factor induced by LPS stimulation that regulates expression of numerous proglycolytic enzymes and proinflammatory cytokines including IL-1β, and its stability is tightly regulated by metabolites of the TCA cycle, such as succinate, fumarate, citrate, and OAA, which inhibit the activity of the HIF-1α prolyl hydroxylases (PHDs) (21). OxPAPC treatment stabilizes HIF-1α activity and potentiates IL-1β production in the presence of an intact TCA cycle (22).

## Consequences of shifts in mitochondrial metabolism

### Effects of metabolites on macrophage activation

**Figure 1:**
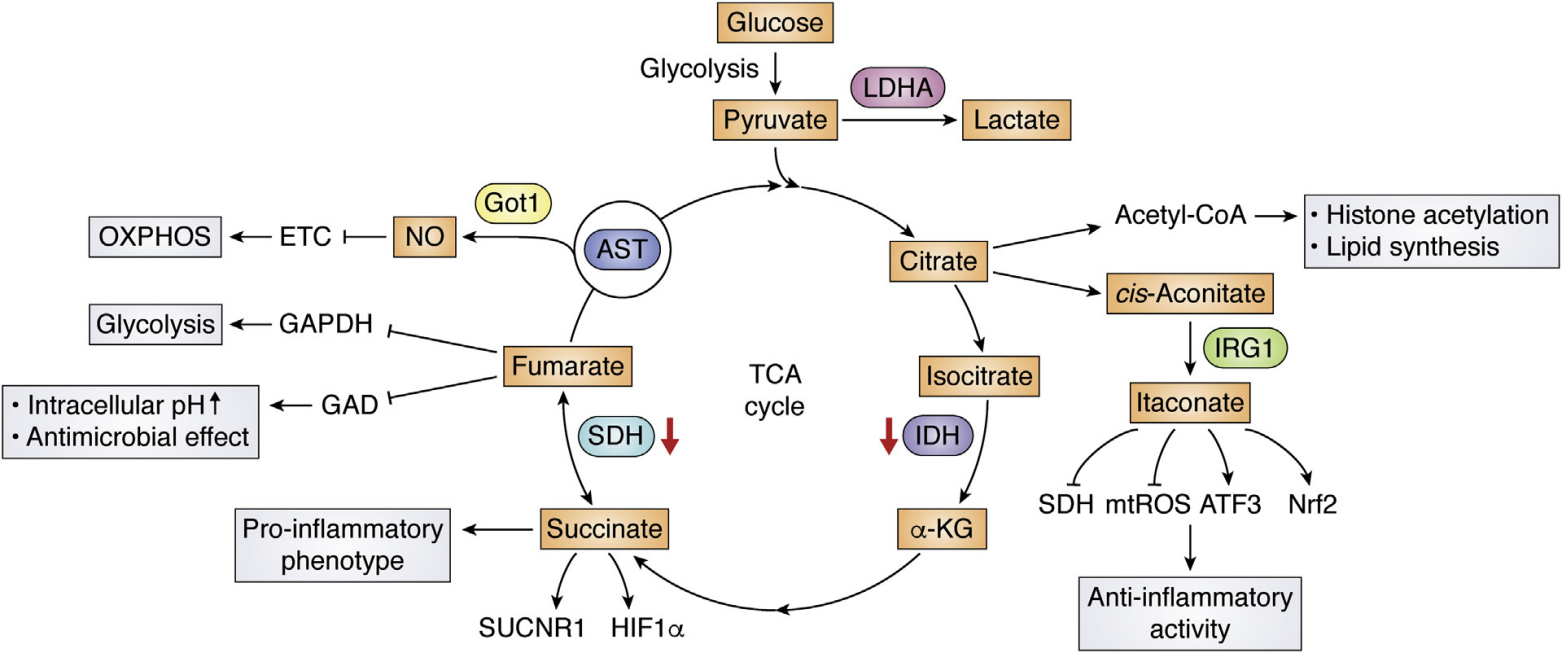
**The effect of TCA intermediates on macrophage activation.** Proinflammatory macrophages exhibit two breaks in the TCA cycle (at IDH and SDH), leading to the accumulation of citrate and succinate, and induction of the arginine-succinate shunt (AST) to support NO production. Itaconate, produced by the enzyme immune-responsive gene 1 (IRG1), exerts anti-inflammatory effects by inhibiting the activity of SDH and stimulating Nrf2 and activating transcription factor 3 (ATF3) induction. Fumarate, another TCA metabolite, is highly antimicrobial toward *L. monocytogenes* under acidic conditions by inhibiting the GAD (glutamic acid decarboxylase) system, which results in intracellular pH increase. It also has an inhibitory effect on aerobic glycolysis by suppressing GAPDH activity.

Shifts in metabolism, including oxidative metabolism, influence the production of various metabolites that have been shown to have powerful roles in influencing inflammation through effects on signaling pathways, transcription factors, and chromatin (23, 24, 25). Because of multiple disruptions to the TCA cycle in macrophages stimulated with LPS, certain metabolites such as citrate, itaconate, and succinate accumulate and play important roles in inflammatory macrophages (Fig. 1).

#### Citrate

Citrate production can be linked to production of a nuclear-cytosolic pool of acetyl coenzyme A (Acetyl-CoA), which serves as a substrate for histone acetylation and lipid synthesis, both of which have been shown to support macrophage activation (26, 27, 28, 29). Citrate is exported from the mitochondria through the mitochondrial citrate carrier (CIC), followed by its cleavage into acetyl-CoA and oxaloacetate by ATP citrate lyase (ACLY) in the cytosol. Acetyl-CoA is necessary for TNFα or IFNγ to induce NO and prostaglandin production and has been shown to fuel histone acetylation at IL-4 and LPS-inducible genes in M2 and M1 macrophages, respectively (27). Oxaloacetate is needed for NO and ROS production by providing NADPH (30). In addition, citrate is acted on by the mitochondrial aconitase 2 (ACO2) to produce *cis*-aconitate, which is further decarboxylated for itaconate synthesis (31). Itaconate acts as a negative regulator of inflammation by inhibiting SDH and the production of the inflammatory cytokines (32). These findings endow citrate, transported from the mitochondria, a crucial role in LPS signaling, *via* effects on production of ROS, NO, prostaglandin, and itaconate as well as effects on inducible histone acetylation and gene expression (33, 34)

#### Itaconate

Itaconate, produced in the mitochondrial matrix from the TCA cycle metabolite *cis*-aconitate by the enzyme immune-responsive gene 1 (IRG1) during LPS stimulation (35, 36), critically regulates multiple aspects of macrophage functions (32). A growing number of studies show that itaconate can exert anti-inflammatory effects by inhibiting inflammatory gene expression and reducing oxidative stress in activated macrophages (32, 37). Itaconate influences oxidative metabolism by suppressing the activity of SDH, a key enzyme in TCA cycle and in Complex II of the ETC. Such activity of itaconate leads to succinate accumulation and decreases in oxygen consumption and contributes to its anti-inflammatory activity (32, 38). Furthermore, itaconate acts as an electrophile to alkylate cysteine residues on KEAP1 protein (37), which is a key oxidative and electrophilic sensor and normally drives ubiquitination and degradation of the anti-inflammatory transcription factor NF-E2–related factor 2 (Nrf2) protein (39), thus allowing newly synthesized Nrf2 to accumulate, translocate into the nucleus, and initiate a transcriptional antioxidant and anti-inflammatory program. In this way, LPS-induced itaconate production leads to Nrf2 protein accumulation and induction of target genes with antioxidant and anti-inflammatory actions (37). In contrast, other studies suggest that effects of itaconate on inflammation are not Nrf2-dependent (40). They reported that electrophilic properties of itaconate and its derivatives inhibit IκBζ protein induction through activating transcription factor 3, leading to selective inhibition of some TLR-inducible transcriptional responses (41). Finally, there may be a negative feedback loop between itaconate and type I interferon signaling. Type I interferons enhance the expression of *Irg1* and the generation of itaconate, but itaconate limits the type I interferon responses by repressing mitochondrial ROS production as well as proinflammatory cytokines, including IL1-β and IL6 (32, 42).

#### Succinate

It has been shown that during inflammatory macrophage activation, the Krebs cycle metabolite succinate accumulates and enhances mitochondrial ROS production, acting as a signal to activate proinflammatory gene expression (43, 44). Succinate oxidation by succinate dehydrogenase (SDH) leads to HIF-1α stabilization through effects on reverse electron transport (RET) and PHD inhibition, leading to the induction of glycolytic genes and sustaining the glycolytic metabolism of inflammatory macrophages (43). The accumulation of succinate is further linked to the induction of a proinflammatory phenotype through autocrine stimulation of a receptor called succinate receptor 1 (SUCNR1) that activates inflammatory pathways by enhancing IL-1β production (45). In intestinal Tuft cells of the gut that express high levels of SUCNR1, succinate has also been shown to activate microbiota-induced type 2 immunity in response to certain infectious agents (46, 47).

#### Fumarate

Fumarate is another TCA metabolite that regulates macrophage functions. Fumarate is highly antimicrobial toward *Escherichia coli* and *Listeria monocytogenes* under acidic conditions, by inhibiting the GAD (glutamic acid decarboxylase) system, which converts glutamate to γ-amino butyric acid (GABA), resulting in intracellular pH increase (48, 49). Dimethyl fumarate (DMF), a derivative of the TCA cycle intermediate fumarate, is used to treat inflammatory diseases such as psoriasis and multiple sclerosis (50). DMF inactivates the catalytic cysteine of the glycolytic enzyme glyceraldehyde 3-phosphate dehydrogenase (GAPDH) to downregulate aerobic glycolysis in activated myeloid and lymphoid cells (50). Moreover, exogenous DMF or endogenous fumarate can modify gasdermin D (GSDMD) at critical cysteine residues to form S-(2-succinyl)-cysteine. Since GSDMD is an executioner of pyroptosis, succination of GSDMD blocks its interaction with caspase 1, decreasing its processing, oligomerization, and capacity to cause cell perforation and pyroptotic cell death (51). A recent study has also found that Nrf2 agonists 4-OI and DMF induce a distinct IFN-independent antiviral program that is broadly effective in limiting virus replication and in suppressing the proinflammatory responses of human pathogenic viruses, including SARS-CoV2 (52). These findings indicate the possibility that fumarate may have a similar effect to DMF in inhibiting aerobic glycolysis and possessing antiviral, antimicrobial, and anti-inflammatory activity.

#### NO

Nitric oxide (NO) is a reactive free radical produced by arginine and catalyzed by nitric oxide synthase (NOS). NO can interact with superoxides to produce reactive nitrogen (RNS), leading to macromolecular changes and cell damage (53). All of these may endow NO produced by macrophage with antibacterial, anti-inflammatory, cytotoxic, and tumoricidal effects (54). A paradox has been found that intracellular NO produced by iNOS plays a proapoptotic role in inflammatory macrophages, but high NO induced by the treatment of LPS and IFN-γ exerts antiapoptotic effect on anti-inflammatory macrophages (55, 56). We proposed that its effect on cell metabolism may attribute to this paradoxical situation. On the one hand, recent labeling analyses indicate that in inflammatory macrophages, part of the TCA cycle is co-opted by the aspartate-arginosuccinate shunt to generate arginine and coordinate NO production. The aspartate-aminotransferase Got1, a key enzyme of the shunt, promotes NO and IL-6 production in inflammatory macrophages, while suppressing mitochondrial respiration (12). NO rapidly triggers release of the mitochondrial-dependent apoptotic mediators, such as cytochrome c, into the cytosol (57). On the other hand, iNOS-derived NO is known to be a vital effector of inflammatory macrophages that adjusts ETC activity by inhibiting critical N-module subunits in Complex I (58) and Complex IV (59). By these means, NO and NO-derived reactive nitrogen species are responsible for inhibiting OXPHOS in stimulated macrophages, thus modulating levels of the essential TCA cycle metabolites citrate and succinate, as well as the anti-inflammatory mediator itaconate (16, 58, 60), resulting in reduced production of inflammatory mediators.

### How oxidative metabolism influences gene expression

#### Histone modifications

Growing evidence supports the notion that oxidative metabolism-derived metabolites converge on chromatin to regulate gene expression, either by providing substrates for histone modification or by influencing the activity of enzymes, which modulate histone modifications. Recent studies indicate that acute LPS exposure augments oxidative metabolism and increases the availability of the TCA cycle intermediate citrate that is used by ATP citrate lyase (ACLY) to produce a nuclear-cytoplasmic pool of Ac-CoA, the carbon substrate for histone acetylation, thus enhancing histone acetylation at inflammatory gene promoters to induce inflammatory responses (28, 61). In contrast, prolonged exposure to LPS drives a shift to oxidative metabolism shutdown in LPS tolerant macrophages, thus reducing histone acetylation at inflammatory genes due to the decreased production of Ac-CoA (28). Therefore, oxidative metabolism influences inflammatory gene induction and suppression *via* effects on Ac-CoA availability for histone acetylation.

TCA cycle intermediates influence the activities of DNA and histone methylation enzymes and shape the epigenetic landscape of chromatin. One example is α-KG, which is a necessary cofactor for some dioxygenases that regulate DNA and histone demethylation, *i.e.*, Ten-Eleven Translocation (TETs) and Jumonji C domain containing (JmjC) demethylases. Upon IL-4 stimulation, α-KG produced *via* glutaminolysis supports the activity of the histone demethylase Jmjd3 to drive loss of trimethylation of histone H3K27, a repressive epigenetic marker, to induce transcription of anti-inflammatory genes (62). Interestingly, succinate and fumarate, also TCA cycle intermediates and structurally related to α-KG, may inhibit α-KG-dependent enzymes. So, the balance of TCA cycle reactions can affect levels of DNA and histone methylation to control gene expression (63, 64). Specifically, succinate and fumarate could increase H3K4me3 levels at the promoters of proinflammatory cytokine genes through the suppression of KDM5 histone demethylases, thus driving gene induction (63, 65, 66).

Apart from histone acetylation and methylation, histone lactylation has been recently implicated in regulating macrophage gene expression. In inflammatory macrophages, reduced oxidative metabolism but increased aerobic glycolysis fuels production of the glycolysis end-product lactate, which is used as a carbon substrate for histone lactylation. Histone lactylation drives a subset of genes induced in M2-like macrophages, particularly homeostatic genes that are involved in wound healing (67).

#### Sirtuin-mediated deacetylation

Sirtuins such as SIRT1 are conserved NAD^(+)^-dependent deacylases and ADP ribosyl transferases (68). It has been reported that SIRT1 suppresses proinflammatory cytokine production by deacetylating p65 at the early stage of LPS stimulation and deacetylates but activates PGC-1β to promote FAO and mitochondrial biogenesis at the late stage of LPS stimulation (69, 70, 71, 72). Furthermore, the Sirt1 signaling cascade can be activated by increased NAD^+^ availability to enhance IL-10 production, thus polarizing macrophages for tissue repair during efferocytosis (73). Another study has also revealed that Sirt1 and Sirt6 are required for the switch from glycolysis to enhanced fatty acid mitochondrial oxidation, while Sirt6 suppresses glucose metabolism by epigenetically silencing the HIF-1α pathway, consequently contributing to a shift toward FAO (74).

#### eIF5A hypusination

Hypusine is uniquely formed through posttranslational modification of a specific lysine residue in eukaryotic translation initiation factor 5A (eIF5A) by the enzymes deoxyhypusine synthase (DHS) and deoxyhypusine hydroxylase (DOHH). Such hypusination occurs shortly after eIF5A synthesis and is needed for eIF-5A activity (75), and according to a recent study, is dynamically regulated after macrophage activation. In IL-4-stimulated macrophages, an increase in production of the polyamine spermidine fuels an increase in eIF5A^H^ to improve the translation efficiency of certain mitochondrial proteins participating in the TCA cycle and OXPHOS, including succinyl-CoA synthetase (SUCLG1) and SDH (76). Another study showed that eIF5A^H^ is vital for inducing the expression of autophagy transcription factor TFEB, thus implicating a role in autophagy regulation (77).

## Shifts in mitochondrial metabolism influence macrophage biology

### Mitochondrial dynamics influence macrophage metabolism and gene expression

**Figure 2:**
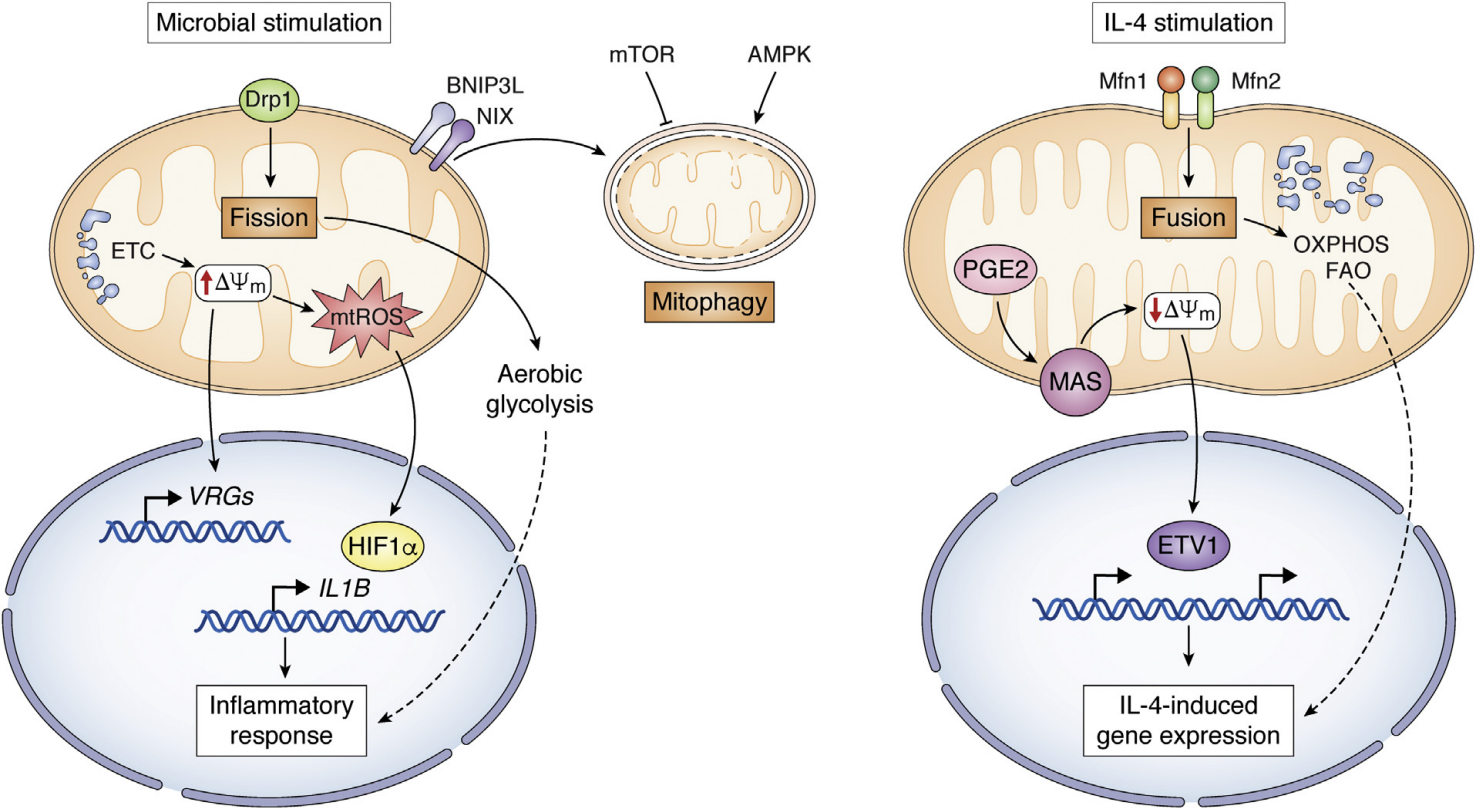
**Mitochondrial metabolism modulates gene expression in inflammatory and IL-4-stimulated macrophages.** In inflammatory macrophages activated by microbial signals, mitochondrial fission dampens ETC efficiency and enhances aerobic glycolysis. Elevated ΔΨm leads to accumulation of mtROS and induction of *Il1b* gene and voltage-regulated genes (VRGs), all contribute to macrophage function. In IL-4-stimulated macrophages, mitochondrial fusion stimulates interactions between ETC complexes that are conducive to OXPHOS and FAO. PGE2 modulates the expression of genes encoding the malate-aspartate shuttle (MAS), leading to the decrease of ΔΨm, which increases the activity of ETS variant 1 (ETV1) to promote some IL-4-inducible gene expression. Degradation or turnover of mitochondria *via* mitophagy is regulated by BNIP3L/NIX receptor and AMPK and mTOR pathway.

Mitochondria represent a major metabolic hub and coordinate metabolic shifts in response to intra- and extracellular signals. Such a role for mitochondria can involve corresponding changes to their morphology and ultrastructure, since nutrient deprivation drives mitochondrial fusion and cristae remodeling to favor coupled respiration and bioenergetic efficiency, while nutrient excess triggers mitochondrial fission and cristae expansion to potentiate uncoupled respiration and reduce mtROS production (78). Mitochondrial fusion in IL-4-stimulated macrophages stimulates interactions between ETC complexes that are conducive to OXPHOS and FAO, while fission in inflammatory macrophages causes cristae expansion to dampen ETC efficiency and enhance aerobic glycolysis (Fig. 2). In TLR-stimulated macrophages, the outer mitochondrial membrane protein FAM73b (also known as Miga2) plays a vital role in switching mitochondria from fission to fusion and thus decreasing IL-12 expression (79). Consistently, mitochondrial fusion regulators Mfn1 and Mfn2 have similar effects on inflammatory gene expression as FAM73b (80, 81). Furthermore, aberrant mitochondrial elongation due to dynamin-related protein 1 (Drp1) knockdown initiates NLRP3-dependent caspase-1 activation and IL-1β secretion, while induction of mitochondrial fission impedes NLRP3 inflammasomal assembly and activation (82).

### Mitochondrial membrane potential exerts effects on macrophage functions

The mitochondrial membrane potential (ΔΨm) is generated by proton pumping at multiple sites of the ETC and is needed for multiple aspects of mitochondrial physiology including uptake/import of mitochondrial matrix proteins and many metabolites and ions (83). ΔΨm can be influenced by the efficiency of the TCA cycle, the availability of NADH and FADH_2_ to feed the ETC, and expression of mitochondrial uncoupling proteins and matrix resident protein such as MICUs, MCUR1, and EMRE (84, 85, 86). It has been pointed out that LPS stimulation leads to an elevated ΔΨm, which together with enhanced SDH oxidation of succinate leads to accumulation of mtROS and promotion of *Il1b* gene induction (44). Moreover, ΔΨm affects the expression of a set of voltage-regulated genes (VRGs) and mediates mitochondria-directed regulation of macrophage function. In IL-4-stimulated macrophage, Prostaglandin E2 (PGE2) modulates the expression of genes encoding the malate-aspartate shuttle (MAS) and reduces levels of MAS metabolites, leading to the decrease of ΔΨm. The reduced ΔΨm increases the activity of a transcription factor called ETS variant 1 (ETV1) to promote the expression of some IL-4-inducible genes (87) (Fig. 2).

### Mitophagy

Degradation or turnover of mitochondria *via* autophagy (mitophagy) is an evolutionarily conserved mechanism for mitochondrial quality control and homeostasis (88). Upon stress or inflammation, mitophagy prevents the accumulation of damaged mitochondria and the increased steady-state levels of ROS that otherwise leads to oxidative stress and cell death. Mitophagy may restrict inflammatory cytokine secretion and directly regulate mitochondrial antigen presentation and immune cell homeostasis. Moreover, the mitophagy receptor BNIP3L/NIX-dependent mitophagy manipulates metabolic reprogramming toward glycolysis, supporting inflammatory macrophage polarization to develop a rapid immune response during inflammation (89). Mitophagy can also be stimulated by the energy sensor AMP-activated protein kinase (AMPK), leading to inactivation of the NLRP3 inflammasome (90). In macrophages, activated mTOR can suppress mitophagy, while selective inhibition of PI3K/Akt/mTOR signaling will lead to the accumulation of dysfunctional mitochondria and induce macrophage apoptosis (91).

## Mitochondrial dysfunction and oxidative stress in the inflammatory response

### Mitochondrial stress

**Figure 3:**
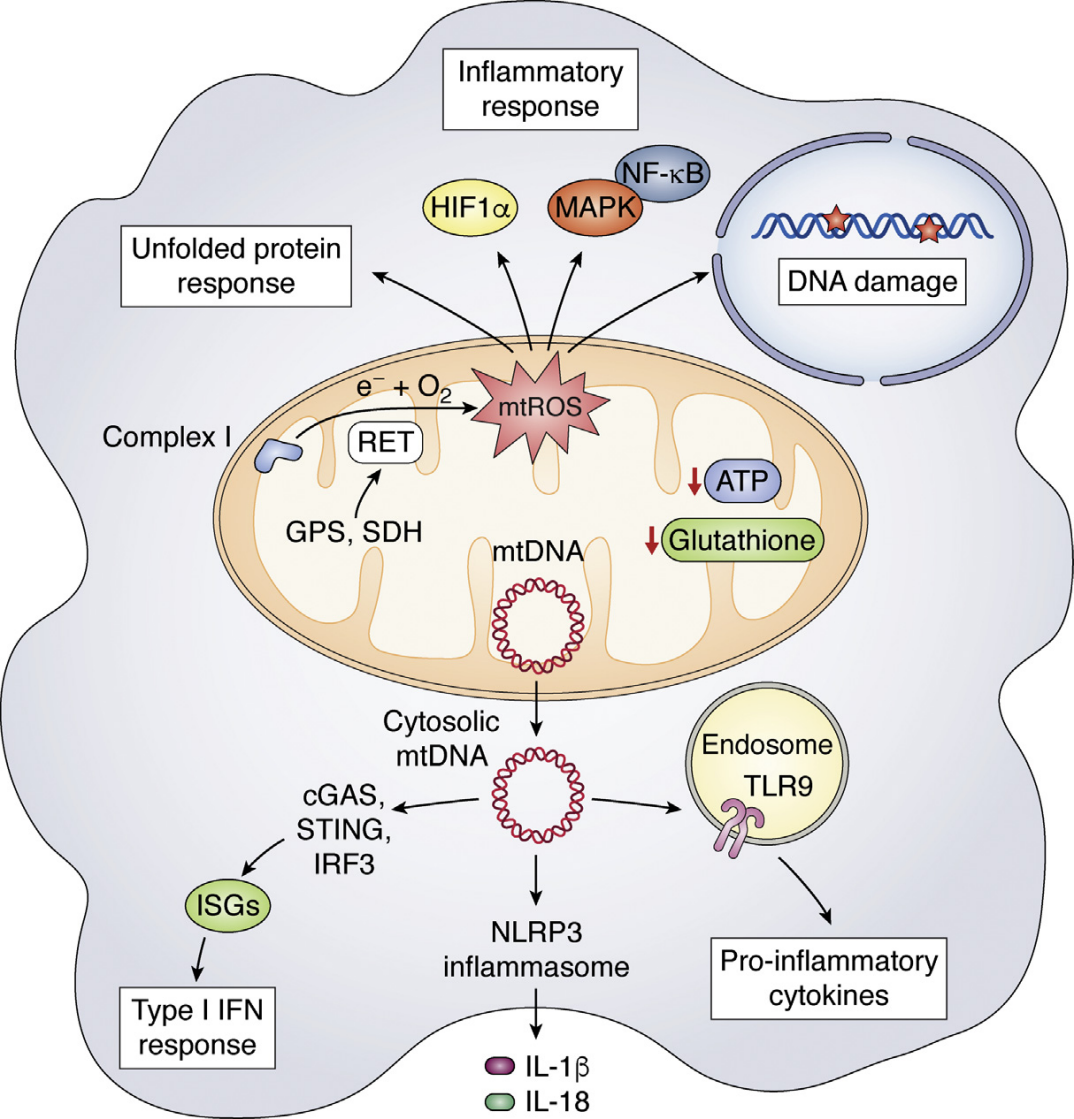
**Mitochondrial stress and mtROS in the inflammatory response.** Mitochondrial dysfunction including mtROS generation, reduced ATP synthesis and glutathione levels, and mtDNA release into the cytosol may all lead to mitochondrial stress. Cytoplasmic mtDNA can stimulate type I IFN responses *via* the cGAS-STING-IRF3 pathway and activate NLRP3 inflammasome and TLR9 pathway to increase proinflammatory cytokines production. mtROS production has been shown to cause DNA damage, unfolded protein response (UPR), and inflammatory responses through HIF-1α and MAPK/NF-κB pathways in LPS-stimulated macrophages.

Mitochondria are one of the main targets of cellular stress induced by inflammation, pathogen infection, and aging. Mitochondrial perturbations including mtROS generation, ATP synthesis reduction, glutathione levels reduction, and mitochondrial morphology alterations may all lead to mitochondrial stress (13, 92) (Fig. 3). In inflammatory macrophages, mitochondrial stress can contribute to release of DAMPs such as mitochondrial DNA (mtDNA) that stimulate innate immune receptors and downstream pathways, implicating mitochondria as both a target and an instigator of the inflammatory response (93). For example, herpesvirus infection induces mtDNA stress and aberrant mtDNA packaging to promote mtDNA escape into the cytosol, where it is recognized by the DNA sensor cyclic GMP-AMP synthase (cGAS) to activate STING (stimulator of interferon genes)-IRF3-dependent signaling and induce interferon (IFN)-stimulated gene (ISG) expression, enhance type I IFN responses, and confer broad viral resistance (93). The mtDNA also can be released into the extracellular plasma, where it activates the TLR9 pathway to increase proinflammatory cytokine production (94, 95). Moreover, mitochondrial stress may cause mitochondrial unfolded protein response and mitophagy to eliminate dysfunctional mitochondria characterized by low membrane potential and a high level of ROS. If all else fails, stressed macrophages undergo apoptosis (96, 97)

Inflammasomes are both a key target and an instigator of mitochondrial stress in its induction of inflammatory responses (98). PAMPs or DAMPs including mtROS and oxidized mtDNA can prime and activate the NLRP3 inflammasome, leading to the secretion of inflammatory cytokines including IL-1β and IL-18 (99, 100) and pyroptosis, a form of lytic programmed cell death (101). On the other hand, it has been reported that blockade of the p38 MAPK signaling pathway represses expression of the NLRP3 inflammasome and IL-1β and cleavage of caspase-1, thus predisposing macrophages to die from noninflammatory apoptosis rather than proinflammatory pyroptosis (102). NLRP3 is modified and activated by acetylation in macrophages and is deacetylated by Sirtuin 2 (SIRT2), a cytosolic NAD^+^-dependent deacetylase and metabolic sensor, contributing to aging-associated inflammation and insulin resistance (103).

### Mitochondrial ROS (mtROS)

ROS, including superoxide (O_2_^−^), hydrogen peroxide (H_2_O_2_), and hydroxyl radical (OH), are very reactive and can attack lipids, proteins, and DNA (104). ROS can be produced at multiple intracellular sites including the mitochondria, endoplasmic reticulum, peroxisomes, and phagosomes; in activated macrophages, NADPH oxidase and mitochondrial ETC activity are thought to be major sources (105, 106). Recently, reverse electron transport (RET) has been shown to contribute to mtROS production in LPS-stimulated macrophages. Increased mitochondrial oxidation of succinate *via* SDH and an elevation of ΔΨm combine to boost RET and consequent mtROS production at Complex I, leading to increased induction of IL-1β (44). Another study reported that the glycerol phosphate shuttle contributes to RET and mtROS production in LPS tolerant macrophages (28). Increased NADH/NAD^+^ ratio in LPS-stimulated macrophages can also favor the generation of O^2−^ at Complex I (107, 108).

How does increased mtROS contribute to macrophage functions? mtROS has been shown to promote killing of phagosomal bacterial by inducing cellular H_2_O_2_ (105). Furthermore, mitochondrial enzyme superoxide dismutase-2 (SOD2) can be delivered from mitochondria to bacteria-containing phagosomes *via* mitochondria-derived vesicles (MDVs), maintaining phagosomal H_2_O_2_ production and thus bacterial killing (109). mtROS can also act as a proinflammatory signal to induce proinflammatory gene expression and cytokines production through regulating MAPK and NF-κB pathways (44, 110, 111). Moreover, mtROS production is implicated in the stabilization of HIF-1α, thus promotes aerobic glycolysis and IL-1β induction in LPS-activated macrophages (112). Finally, mtROS produced by Complex III of the mitochondrial ETC plays a role in the DNA damage response and NAD^+^ metabolism in LPS-stimulated macrophages. Such mtROS promotes DNA damage and activation of DNA damage-sensing poly (ADP-ribose) polymerases (PARPs), leading to depletion of its substrate NAD^+^. Consequent induction of nicotinamide phosphoribosyl transferase (NAMPT), a key enzyme in the NAD^+^ salvage pathway, is linked to maintenance of glycolytic flux, Warburg metabolism, and the inflammatory response (113). The increased level of mtROS results from inhibited glycolytic activity, contributing to exacerbated unfolded protein response (UPR) and inflammatory response (114).

Altogether, these findings implicate mtROS as an important component of antibacterial responses and inflammatory cytokine production and further establish the critical role of mitochondria in regulating innate immune signaling in macrophages.

## Conclusions and perspectives

Mounting evidence indicates that mitochondrial metabolism coordinates signal transduction, chromatin regulation, and transcriptional regulation to influence macrophage activation and fine-tune the immune responses. Mitochondria can no longer be viewed solely as the energy machinery of the cell but as a vital source of dynamic signals that coordinate changes to shifting environments. Future work may focus on the adaptive and dynamic mitochondrial responses of macrophages in other settings. For example, tissue resident macrophages (TRMs) reside in most tissues of the body, where they are thought to serve as critical support cells with context-dependent roles in maintaining tissue homeostasis and tissue stress adaptation. Some studies are starting to report how macrophage metabolism influences such roles (115), but information is currently limited due to technological challenges in examining mitochondrial metabolism *in situ* and/or from small numbers of TRMs. With technological advances in metabolite profiling and other metabolic techniques on the horizon (116), we can expect to see some breakthroughs in our understanding of the role of TRMs mitochondrial metabolism in the future.

## Conflict of interest

The authors declare that they have no conflicts of interest with the contents of this article.
